# Modelling The Combined Effects Of Collagen and Cyclic Strain On Cellular Orientation In Collagenous Tissues

**DOI:** 10.1038/s41598-018-26989-y

**Published:** 2018-06-04

**Authors:** T. Ristori, T. M. W. Notermans, J. Foolen, N. A. Kurniawan, C. V. C. Bouten, F. P. T. Baaijens, S. Loerakker

**Affiliations:** 1 Department of Biomedical Engineering, Eindhoven University of Technology, PO Box 513, 5600 MB Eindhoven, The Netherlands; 2 Institute for Complex Molecular Systems, Eindhoven University of Technology, PO Box 513, 5600 MB Eindhoven, The Netherlands

## Abstract

Adherent cells are generally able to reorient in response to cyclic strain. In three-dimensional tissues, however, extracellular collagen can affect this cellular response. In this study, a computational model able to predict the combined effects of mechanical stimuli and collagen on cellular (re)orientation was developed. In particular, a recently proposed computational model (which only accounts for mechanical stimuli) was extended by considering two hypotheses on how collagen influences cellular (re)orientation: collagen contributes to cell alignment by providing topographical cues (contact guidance); or collagen causes a spatial obstruction for cellular reorientation (steric hindrance). In addition, we developed an evolution law to predict cell-induced collagen realignment. The hypotheses were tested by simulating bi- or uniaxially constrained cell-populated collagen gels with different collagen densities, subjected to immediate or delayed uniaxial cyclic strain with varying strain amplitudes. The simulation outcomes are in agreement with previous experimental reports. Taken together, our computational approach is a promising tool to understand and predict the remodeling of collagenous tissues, such as native or tissue-engineered arteries and heart valves.

## Introduction

Cellular orientation greatly influences the *in vivo* mechanical properties and functionality of soft tissues, such as arteries and heart valves. In fact, primarily along their main direction, cells exert traction forces^1^ capable of deforming soft tissues, and they secrete collagen^2–4^, which is the main load-bearing component of most of these tissues. Therefore, predicting and understanding cellular (re)orientation is of great importance for understanding soft tissue mechanics and remodeling.

Cells in collagenous tissues can change their orientation in response to a wide range of stimuli^5,6^. For example, Guido and Tranquillo^7^ have demonstrated that cells tend to align along the topographical cues provided by collagen, a phenomenon known as contact guidance^8^. Numerous studies have also shown that cells seeded in differentially constrained collagenous constructs align in the direction of the constraints, as a result of stiffness anisotropy^9–13^. Finally, it has been observed that cells seeded in biaxially constrained collagenous tissues that are uniaxially and cyclically strained generally align perpendicular to this mechanical stimulus^12,14,15^. However, extracellular collagen fibers can affect the reorientation potential of cells in response to cyclic strain, a phenomenon that depends on the density of collagen, as shown by Foolen and colleagues^12,15^. In their studies, they analysed biaxially constrained cell-populated collagen gels that were statically cultured for 3 days followed by 3 days of uniaxial cyclical strain, and they demonstrated that cells in the inner layer of the tissues could reorient perpendicular to cyclic strain only in case of a relatively low collagen seeding density (0.45 mg/mL), while they did not reorient in relatively high-density collagen gels (1.5 mg/mL). The mechanisms responsible for these outcomes are not fully understood yet and further studies are therefore needed.

In this context, computational models are a valuable tool because of their predictive potential and efficiency in testing hypotheses. Several models to predict the (re)orientation of adherent cells have been proposed^16–26^ but, if adopted to simulate the experiments described in the previous paragraph, they could not predict the observed combined effects of cyclic strain and collagen on cellular (re)orientation. For example, Barocas and Tranquillo^16^ have developed a theoretical model to explain the remodeling of collagenous tissues and the (re)orientation of cells and collagen. Similar to other studies^17,22,25^, they assumed that cells co-align with collagen because of contact guidance, irrespective of mechanical cues which, in turn, affect the orientation of collagen fibers. It has been demonstrated that this model can predict the remodeling of collagenous tissues under static conditions, with different boundary conditions^27,28^. More recently, Rouillard and Holmes^26^ developed an agent-based model to study the remodeling of collagen and cells in healing infarcts. In their study, both the effects of mechanical stimuli and collagen orientation were considered to predict the alignment of cells. Both the theoretical model of Barocas and Tranquillo^16^ and the agent-based model of Rouillard and Holmes^26^ could be adopted to analyse the experiment of Foolen *et al*.^12,15^ described in the previous paragraph. However, these two cited models do not consider the influence of strain rate on cellular reorientation and, due to this limitation, they could not predict the experimentally observed strain avoidance response of cells in response to uniaxial cyclic strain in biaxially constrained collagenous tissues^12,15^. Recently, Obbink-Huizer *et al*.^29^ have developed a computational model for cellular reorientation in response to mechanical stimuli, built upon the frameworks of Deshpande *et al*.^30^ and Vernerey and Farsad^31^. This computational model is based on the assumption that cells co-align with their stress fibers (SFs), which remodel in response to the experienced strain and strain rate. In addition to explaining SF remodeling of cells on two-dimensional substrates, this model can predict cellular alignment in three-dimensional tissues. Yet, it does not consider the possible influence of collagen on the response of cells to cyclic strain, and therefore cannot predict and explain cellular reorientation when collagen effects override mechanical stimuli^12,14,15^.

**Figure 1:**
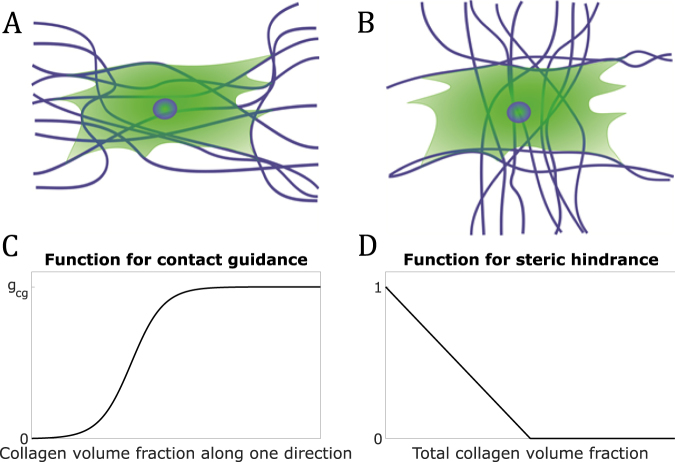
Scheme of hypotheses (**A**,**B**) and functions introduced to model them (**C**,**D**). Cells might not react to cyclic strain because (**A**) they locally follow the direction of collagen, according to contact guidance, or (**B**) they do not have space to reorient due to collagen, according to steric hindrance. (**C**) Function describing the effects of contact guidance. (**D**) Function describing the effects of steric hindrance.

In this study, to overcome the limitations of previous computational models and improve our understanding of the interplay between cells and collagen, we extended the model of Obbink-Huizer *et al*.^29^ by including the effects that collagen networks can have on cellular (re)orientation. Two possible hypotheses were considered and implemented separately: collagen contributes to cell alignment by providing topographical cues (hypothesis of contact guidance, Fig. 1A); collagen causes a spatial obstruction for cellular reorientation (hypothesis of steric hindrance, Fig. 1B). In addition, to capture the reciprocal interactions between cells and the surrounding collagen fibers, we developed an evolution law for the rearrangement of collagen induced by cellular forces. In what follows, we first describe the proposed approach to model collagen reorganization and the effects that collagen can have on SF remodeling and cellular reorientation. Then, we test our two hypotheses by simulating the reorganization of cell-populated collagen gels that were biaxially or uniaxially constrained, and subjected to immediate or delayed uniaxial cyclic stretch. For a complete overview of the equations used to simulate the remodeling of these collagen gels, we refer the reader to the supplementary information.

## Results

### Proposed law for the rearrangement of unstable collagen networks

Experimental studies have shown that collagen networks can be susceptible to reorganization driven by cellular forces^3,12,32–34^. Hereafter, we use the term “unstable” to describe collagen networks that can be rearranged by cellular forces, and “collagen volume fraction along a direction” as the fraction of the total material along that specific direction consisting of collagen fibers. For the collagen rearrangement law, we hypothesized that, because of cellular forces, unstable collagen fibers are reoriented from their original orientation towards the directions with higher cellular tension. This was not modelled by direct rotation of the fibers, but was modelled through removal and replacement of fibers in specific fiber directions, which were rotated only based on affine kinematics. Thus, only the volume fractions of collagen in the different fiber directions changed with time, not the directions themselves. In particular, we assumed that the collagen distribution tends to a preferred one that is equal to the distribution of SF stresses. That is to say

1 $$\frac{{\phi }_{cf,p}^{i}}{\sum _{i=1}^{N}{\phi }_{cf,p}^{i}}=\frac{{\sigma }_{sf}^{i}}{\sum _{i=1}^{N}{\sigma }_{sf}^{i}}$$

where ${\phi }_{cf,p}^{i}$ is the preferred collagen fiber volume fraction along direction $i=1,\mathrm{...},N$(with $N$ the number of fiber directions considered), and ${\sigma }_{sf}^{i}$ denotes the magnitude of the SF stress along that direction. Furthermore, we assumed that the current collagen volume fraction ${\phi }_{cf}^{i}$ tends to the preferred ${\phi }_{cf,p}^{i}$ according to the first-order evolution law

2 $$\frac{d{\phi }_{cf}^{i}}{dt}=\frac{1}{{\tau }_{cr}}({\phi }_{cf,p}^{i}-{\phi }_{cf}^{i})$$

where the parameter ${\tau }_{cr}$ characterizes the rate of collagen reorientation. Both the preferred ${\phi }_{cf,p}^{i}$ and current collagen volume fractions ${\phi }_{cf}^{i}$ were assumed to sum to a constant total collagen volume fraction ${\varphi }_{cf}=\sum _{i=1}^{N}{\phi }_{cf,p}^{i}=\sum _{i=1}^{N}{\phi }_{cf}^{i}$.

### Extending the evolution law for SF remodelling

The evolution law for SF remodeling proposed by Obbink-Huizer *et al*.^29^ can be written as:

3 $$\frac{d{\phi }_{sf}^{i}}{dt}={f}_{mech}({\varepsilon }^{i},{\dot{\varepsilon }}^{i}){\phi }_{m}-{k}_{d}{\phi }_{sf}^{i}$$

Here, ${\phi }_{sf}^{i}$ represents the SF volume fraction along direction $i$ and ${\phi }_{m}$ the volume fraction of monomeric actin. The function ${f}_{mech}({\varepsilon }^{i},{\dot{\varepsilon }}^{i})$ characterizes the SF formation rate dependent on the strain ${\varepsilon }^{i}$ and strain rate ${\dot{\varepsilon }}^{i}$ experienced by the SFs along the *i*-th direction (see supplementary information for more details, equations (S5-S11)), while ${k}_{d}$ describes the rate of SF disassembly. In the present study, equation (3) was extended with the inclusion of the effects that stable and high-density collagen networks can have on SF remodeling. The enhancements were based on the two aforementioned hypotheses, which were implemented separately and are treated below.

The first hypothesis, contact guidance, is motivated by experimental studies that have shown that cells are able to respond to topographical cues by (re)adjusting their orientation. For example, it has been observed that cells cultured on microgrooved substrates tend to align along the direction of these grooves^35–37^ and that these patterns can restrict the reorientation capacity of cells in response to cyclic strain^38–40^. Collagen seems to provide cells with analogous stimuli^7,14,41,42^. Therefore, we hypothesized that collagen provides cells with topographical cues, such that SFs tend to align along directions with higher collagen densities (Fig. 1A). To model this phenomenon, we adapted equation (3) by assuming that higher collagen volume fractions along specific directions induce more SF formation. In particular, we described SF remodeling as

4 $$\frac{d{\phi }_{sf}^{i}}{dt}=({f}_{mech}({\varepsilon }^{i},{\dot{\varepsilon }}^{i})+{f}_{cg}({\phi }_{cf}^{i})){\phi }_{m}-{k}_{d}{\phi }_{sf}^{i}$$

where

5 $${f}_{cg}({\phi }_{cf}^{i})={g}_{cg}(1-\frac{{h}_{1}}{\exp ({h}_{2}{\phi }_{cf}^{i})+{h}_{1}-1})$$

is a monotonically increasing function dependent on ${\phi }_{cf}^{i}$ that describes the increase of SF formation for increasing collagen content along the $i$-th direction (Fig. 1C). In equation (5), ${g}_{cg}$ represents the maximum effect of contact guidance on SF remodeling, while ${h}_{1}$ and ${h}_{2}$ are parameters characterizing the inflection point and the associated slope of the function ${f}_{cg}$. The inflection point is inversely proportional with ${h}_{2}$ and it increases with increasing values of ${h}_{1}$. On the other hand, the slope is directly proportional with ${h}_{2}$ and it decreases for increasing values of ${h}_{1}$.

The second hypothesis, steric hindrance, is motivated by experimental studies that have highlighted that high-density collagen environments can constitute a spatial obstruction for cell migration^43–45^. We propose that a similar mechanism occurs with respect to cellular reorientation and SF remodeling, such that the SF remodeling potential decreases with increasing collagen density and is completely inhibited when a certain threshold density is exceeded (Fig. 1B). This mechanism was modelled by multiplying the term at the right-hand side of equation (3) with a monotonically decreasing function that depends on the total collagen volume fraction ${\varphi }_{cf}$ (Fig. 1D). Specifically, considering the effects of steric hindrance without contact guidance, SF remodeling is described by

6 $$\frac{d{\phi }_{sf}^{i}}{dt}=({f}_{mech}({\varepsilon }^{i},{\dot{\varepsilon }}^{i}){\phi }_{m}-{k}_{d}{\phi }_{sf}^{i}){f}_{sh}({\varphi }_{cf})$$

with

7 $${f}_{sh}({\varphi }_{cf})=\{\begin{array}{cc}1-{g}_{sh}{\varphi }_{cf} & {\rm{if}}\,{\varphi }_{cf} < 1/{g}_{sh},\\ 0 & {\rm{if}}\,{\varphi }_{cf}\ge 1/{g}_{sh}.\end{array}$$

Here, the parameter ${g}_{sh}$ describes the decrease of the rate of SF remodeling for increasing total collagen volume fraction.

### Simulation of biaxially constrained cell-seeded collagen gels undergoing uniaxial cyclic stretch

To test the proposed hypotheses, we simulated the remodeling of biaxially constrained cell-populated collagen gels cultured for 6 days, undergoing immediate or delayed (for 3 days) uniaxial cyclic stretch, and we compared the computational results with previous experimental reports^12,15^. For these tissues, we assumed that collagen networks are unstable during the first 3 days and, due to instability, during this period they can be rearranged by cellular forces but they cannot influence cellular orientation via contact guidance or steric hindrance. Nevertheless, collagen networks in collagen gels become stable over time due to non-covalent interactions^46^ and cellular entanglement^47^. For these simulations, the collagen network organization was assumed to reach a stable configuration after 3 days of remodeling. Thus, from day 3 on, collagen networks provide cells with contact guidance or steric hindrance, and they cannot be further reorganized.

**Figure 2:**
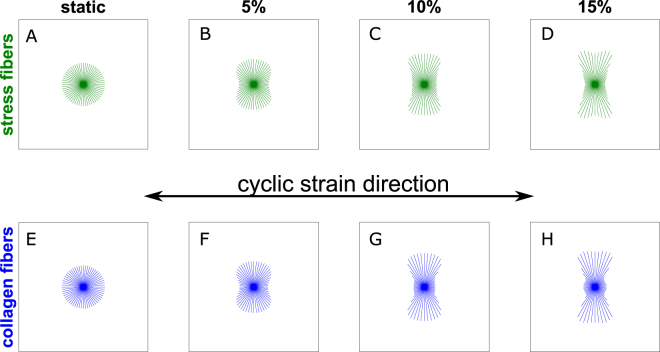
Computational prediction of SF and collagen fiber distributions in biaxially constrained collagen gels cultured for 3 days. The computational simulations predicted that the degree of anisotropy of the SF (**A**–**D**) and collagen (**E**–**H**) distributions is proportional to the amplitude of the horizontally applied cyclic stretch (increasing from left to right). In particular, the distributions are isotropic in case of static constraint (0% stretch, **A** and **E**) and increasingly aligned perpendicular to the applied cyclic stretch (**B**,**C** and **F**–**H**) for increasing stretch amplitudes (5%, 10%, and 15%, reported on top of the graphs). The length of the lines is proportional to the amount of SFs or collagen along that particular direction.

Figure 2 shows SF and collagen orientation after the first 3 days of culture, when the effects of contact guidance and steric hindrance are still absent, under different magnitudes of uniaxial cyclic stretch (0–15%). The simulations predicted that collagen fibers and SFs have an isotropic distribution under static conditions (no external strain applied, Fig. 2A). With uniaxial cyclic strain, due to the absence of collagen effects on cellular orientation, SFs can freely reorient perpendicular to this mechanical stimulus, as governed by equation (3), with the level of anisotropy increasing with the cyclic stretch magnitude (Fig. 2A–D). As cells remodel their cytoskeleton, they exert traction forces and thereby rearrange the collagen network surrounding them. This was well captured by the evolution law for collagen reorientation (Fig. 2E–H), which predicted a collagen fiber orientation perpendicular to the mechanical stimuli, in agreement with experiments^12^.

**Figure 3:**
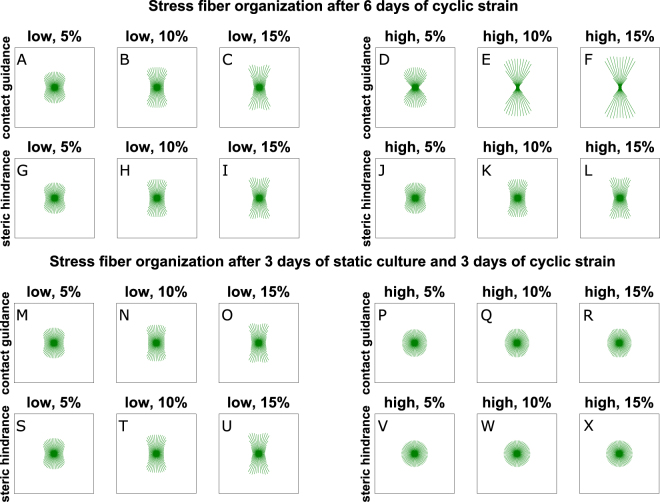
Computational prediction of SF distributions for biaxially constrained collagen gels after 6 days of culture. The tissues were cyclically stretched immediately (**A**–**L**), or after 3 days of static constraint (**M**–**X**). Tissues with relatively low (**A**–**C**, **G**–**I**, **M**–**O**, **S**–**U**) and high (**D**–**F**, **J**–**L**, **P**–**R**, and **V**–**X**) collagen densities were simulated. The cyclic stretch was horizontal with respect to the figure, with increasing amplitude from left to right. The computational simulations predicted that SFs align perpendicular to the applied cyclic stretch in case of immediate stretch (**A**–**L**) and in low-density collagen gels (**M**–**O** and **S**–**U**). Conversely, SFs are isotropic in high-density collagen gels statically constrained for 3 days and cyclically stretched for additional 3 days (**P**–**R** and **V**–**X**).

Next, we tested the hypotheses by simulating 6 days of culture, with either immediate or delayed cyclic straining (Fig. 3). For these simulations, the parameter values ${g}_{cg}=2{s}^{-1}$, ${h}_{1}=500$, and ${h}_{2}=300$ were used for equation (5), ${g}_{sh}=2$ for equation (7), and the remaining parameters were chosen following previous studies (Table S1). When cyclic stretch was applied immediately after seeding (Fig. 3A–L), collagen did not have significant effects on SF orientation, and both proposed models predicted a SF organization perpendicular to cyclic strain, with increasing degree of anisotropy for increasing strain amplitude. In case of contact guidance, this is because the topographical cues provided by the anisotropic collagen architecture predicted for the first 3 days (Fig. 2F–H) were not in competition with the mechanical stimuli and actually slightly enhanced the anisotropy of the SF organization, both for low and high collagen densities, as can be seen by comparing Fig. 2B–D with Fig. 3A–F. In case of steric hindrance, we hypothesized that higher densities of collagen slow down the SF remodeling (equation (6)), but the equilibrium SF configuration was already reached after 3 days (when no collagen effects were present) and in case of 6 days of immediate cyclic strain cells were always subjected to the same mechanical stimuli. Thus, slowing down SF remodeling did not have significant effects (compare Fig. 2B–D with Fig. 3G–L).

When uniaxial cyclic strain was applied after 3 days of static constraint, cells were exposed to a different mechanical environment compared to the first period that had resulted in an isotropic distribution (Fig. 2A). For low collagen density (Fig. 3M–O,S–U), the simulations performed with both extended models predicted that cells can respond to this change by aligning perpendicular to the strain, with the degree of anisotropy increasing with the strain amplitude. Conversely, when the collagen density is relatively high (Fig. 3P–R,V–X), both computational models predicted that cells are not able to respond to the change of mechanical stimulation, and the SF orientation remains isotropic. The predicted distributions have different explanations. On the one hand, with contact guidance, SFs do not respond to mechanical stimuli because these cues are dominated by the topographical stimuli provided by the high-density collagen network, which is isotropic (Fig. 2A). On the other hand, with steric hindrance, SFs cannot respond to mechanical stimuli because their remodeling is inhibited due to lack of space. Therefore, the configuration after 6 days is the same as the configuration after 3 days.

### Comparison with previous experimental results

**Figure 4:**
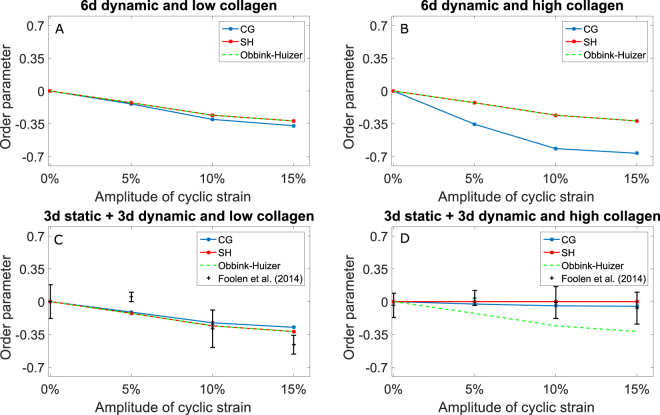
The extended models are quantitatively in agreement with previous experimental data, in contrast to the original model of Obbink-Huizer *et al*.^29^ that cannot predict SF alignment in biaxially constrained high-density collagen gels statically constrained for 3 days and cyclically stretched for additional 3 days. The figures show a comparison of the order parameters of the SF distributions in biaxially constrained collagen gels, as predicted by the two extended computational models (blue for contact guidance, red for steric hindrance) and the original model of Obbink-Huizer *et al*.^29^ (green), with low (**A**,**C**) and high (**B**,**D**) collagen densities. When available in the literature^15^, previous experimental results were reported by showing the mean order parameter and the standard deviation (black).

To compare the computational predictions with the previous experimental results (when available), each SF distribution was quantified with the order parameter used in Foolen *et al*.^15^:

8 $${O}_{sf}=\frac{{\int }_{-\pi /2}^{\pi /2}{\phi }_{sf}(\gamma )\cos (2\gamma )d\gamma }{{\int }_{-\pi /2}^{\pi /2}{\phi }_{sf}(\gamma )d\gamma }$$

where ${\phi }_{sf}(\gamma )$ is the SF volume fraction oriented at an angle $\gamma$ from the direction of applied cyclic strain. The denominator was introduced to normalize this quantity to get the fiber fraction along an angle *γ*. This order parameter is equal to +1 when all SFs are oriented parallel to the strain, −1 when all fibers are aligned perpendicularly, and 0 for SF distributions symmetric with respect to the angles ±45°. In addition, to highlight the effects of the hypotheses of contact guidance and steric hindrance on SF remodeling, the simulations described in the previous section were repeated with the original model of Obbink-Huizer *et al*.^29^. Figure 4 compares the order parameters obtained with the original and extended models, and the experimental results of Foolen *et al*.^15^. The graphs confirm that, in case of immediate stretching (Fig. 4A,B) or low collagen density (Fig. 4A,C), collagen has no significant effects on SF remodeling, since the computational results of the extended models (solid lines) are qualitatively equivalent to the results of the original model (dashed line). In addition, it can be observed that the original and extended models are quantitatively close and qualitatively in agreement with the experimental results (symbols) obtained for low-density collagen gels with delayed cyclic strain and low collagen density (Fig. 4C). In contrast, Fig. 4D shows that, for delayed cyclic strain and high collagen density, the extended models are both qualitatively and quantitatively in agreement with experiments, while the original model was not able to capture the influence of collagen on cellular reorientation.

### Sensitivity analysis

**Figure 5:**
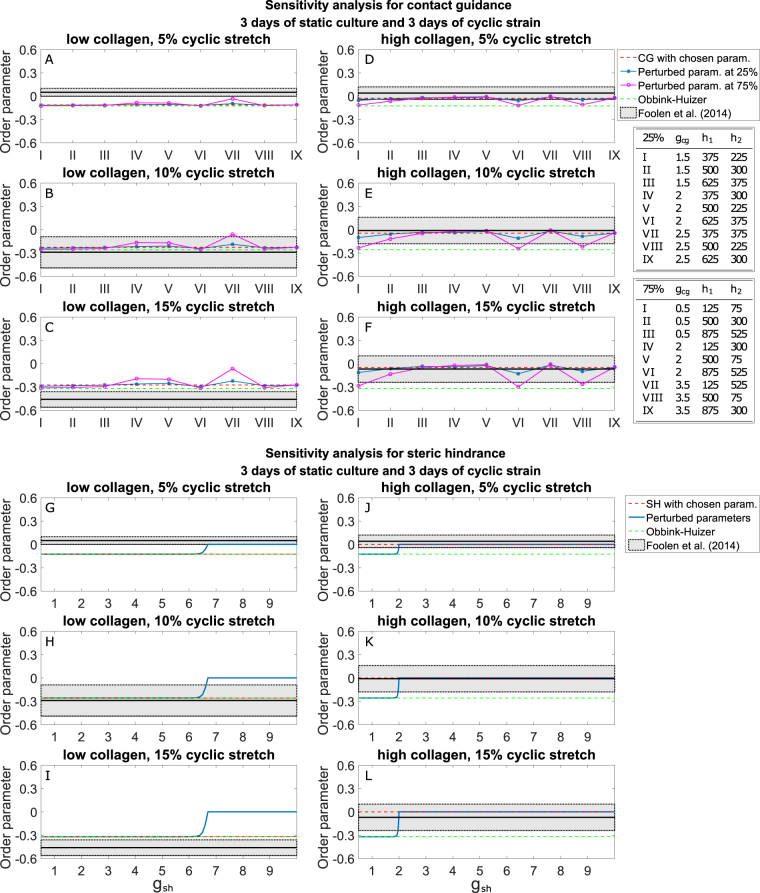
The simulations with contact guidance or steric hindrance are not significantly affected by relatively small variations of parameters, while very large variations can lead to computational results not in agreement with experiments. The figure shows the sensitivity analysis for the order parameter of the SF distributions predicted for biaxially constrained collagen gels cultured at static constraint for 3 days and undergoing uniaxial cyclic stretch for subsequent 3 days, with the hypotheses of contact guidance (**A**–**F**) and steric hindrance (**G**–**L**). In the graphs, the shaded region represents the range of values centred in the mean (black line) of order parameters observed in the previous experiments^15^, with a band width twice the standard deviation. The roman numbers used for the graphs for contact guidance (**A**–**F**) correspond to the perturbations characterized in the legends on the right-side.

Given the similarities of both extended computational models with the experimental results of biaxially constrained collagen gels subjected to uniaxial cyclic strain after static constraint, we performed a sensitivity analysis to determine how much of this conformity is dependent on the choice of parameters. The sensitivity analysis for the contact guidance hypothesis was executed by changing the chosen parameters according to the Taguchi method^48^. Each variation of parameters corresponded to a different SF organization and, consequently, a different value for ${O}_{sf}$. Figure 5A–F display the results of the sensitivity analysis for the model with contact guidance for a 25% and a 75% variation of the parameters used to obtain the results shown in Figs 2–3. In general, the results of the model with contact guidance were qualitatively stable with respect to parameter variations. Only 14 sets of variations out of 108 modified the computational results such that the associated ${O}_{sf}$ was not within the experimental range of results anymore (or vice versa). Among those 14 perturbations, 11 corresponded to the 75% variations. Therefore, the sensitivity analysis indicated that the contact guidance model is rather insensitive even to relatively large variations of the parameters.

The sensitivity analysis for the model with the steric hindrance hypothesis was performed by sampling the single parameter present in this model, and varying its values between 0.5 and 10. Figure 5G–L show that the computational results were consistent for all values of this parameter between 2 and 6. Values smaller than 2 led to a steep increase of anisotropy of the SF organization resulting from the simulations with high collagen density. Similarly, values larger than 6 caused a modification of the results with low collagen density towards more isotropic SF organizations.

In conclusion, the results from both models are not significantly affected by relatively small variations of parameters, while very large variations can lead to computational results that are not qualitatively in agreement with the experimental ones.

### Simulation of uniaxially constrained cell-seeded collagen gels undergoing uniaxial cyclic stretch

**Figure 6:**
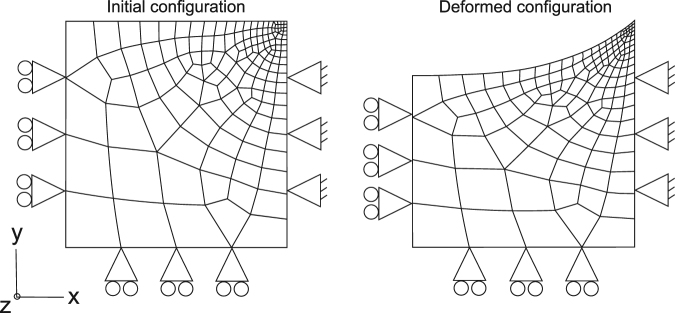
Initial and deformed configurations of one fourth of the uniaxially constrained collagen gel. Cells pull on their surroundings and, consequently, the collagen gel is contracted along its free direction.

**Figure 7:**
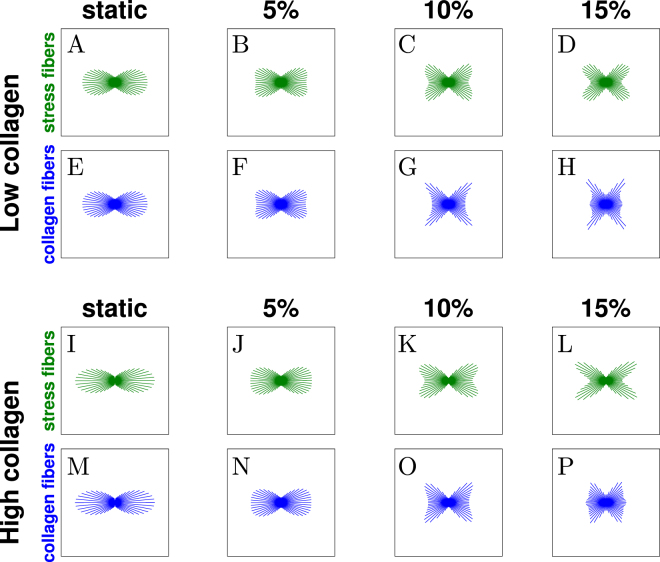
Computational prediction of SF and collagen distributions predicted for uniaxially constrained collagen gels cultured for 3 days. The computational simulations predicted that the SF and collagen distributions are strongly aligned in the constrained direction (horizontal direction) in case of static constraint (0% stretch, **A**,**E**,**I**, and **M**). In case of external cyclic stretch applied in the constrained direction, the alignment of both SFs (**B**–**D** and **J**–**L**) and collagen (**F**–**H** and **N**–**P**) decreases when the stretch amplitude increases (from left to right).

Given that both the models with contact guidance and with steric hindrance successfully predicted the remodeling occurring in biaxially constrained cell-populated collagen gels, the hypotheses were further tested by simulating cell-seeded collagen gels that were only uniaxially constrained, and undergoing immediate or delayed (for 3 days) cyclic strain. These tissues are characterized by heterogeneous collagen and stress fiber distributions. Given that in previous experimental and computational studies the cellular and collagen orientations were analysed only in the centre of the tissues^12,29^, we decided to focus on the alignments in this central portion as well, corresponding to the bottom-left element of the modelled collagen gels (Fig. 6). Again, due to the assumption that collagen networks are initially unstable and have negligible effects on SFs, the results of the two computational models after the first 3 days are identical, as reported in Fig. 7. From this figure, it is apparent that the collagen density did not qualitatively influence the SF distributions, even though small quantitative differences are visible when comparing the results for collagen gels with relatively low (Fig. 7A–H) and high collagen densities (Fig. 7I–P). In both cases, under static conditions, the simulations predicted collagen and SF orientations strongly aligned towards the direction of the tissue constraint (Fig. 7A,E,I and M). This occurs because, under these conditions, cells pull on their surroundings causing compaction of the collagen gel along the unconstrained direction, as shown in Fig. 6. Consequently, the SF stress in the unconstrained direction reduces (equations (S.4) and (S.6)), thereby biasing the orientation of SFs and collagen in the constrained direction.

Immediate cyclic stretching decreased the degree of alignment of both collagen and SFs towards the constrained direction, with this decrease dependent on the amplitude of the applied cyclic stretch (Fig. 7B–D,F–H, J–L and N–P). In these conditions, SFs tend to avoid both the direction of tissue compaction and the direction of cyclic strain. As a result, increasing the cyclic stretch amplitude causes a higher alignment of SFs towards directions in between the compaction direction and the cyclic stretch direction, eventually leading to a cross-like organization for the maximal stretch amplitude (Fig. 7D,L). Similar to static conditions, also for dynamic conditions the simulations predicted that collagen is reoriented until it obtains an architecture comparable with that of the SFs (Fig. 7F–H,N–P).

**Figure 8:**
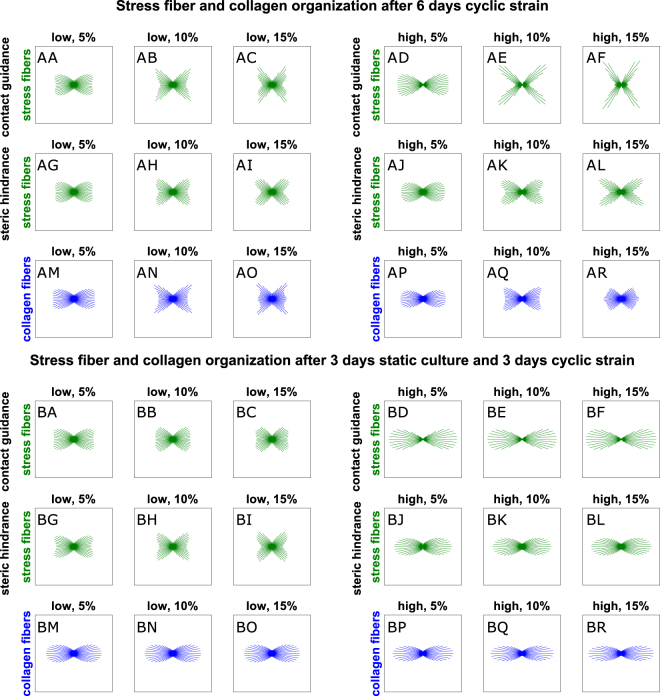
Computational prediction of SF and collagen distributions for uniaxially constrained collagen gels cultured for 6 days. The SF distributions are shown in green (AA-AL and BA-BL), while the collagen distributions are represented in blue (AM-AR and BM-BR). The tissues were cyclically stretched immediately (AA-AR), or after 3 days of static constraint (BA-BR). Tissues with relatively low (AA-AC, AG-AI, AM-AO, BA-BC, BG-BI, and BM-BO) and high (AD-AF, AJ-AL, AP-AR, BD-BF, BJ-BL, and BP-BR) collagen densities were simulated. The cyclic strain was horizontal with respect to the figure, with increasing amplitude from left to right.

Figure 8 shows the computational results for uniaxially constrained collagen gels cultured for 6 days. In this case, some of the predicted SF organizations are cross-like and this leads to values of ${O}_{sf}$ not completely representing the SF distributions. Therefore, we decided to show the order parameter only in the supplementary information (Supplementary Fig. S3) and focus here directly on the distribution of fiber orientations.

For collagen gels subjected to uniaxial cyclic strain for 6 days, the collagen orientation (Fig. 8AA–AR) was predicted to be very similar to results at day 3 (Fig. 7E–H,M–P). These similarities can be explained by the fact that the collagen network was hypothesized to be stable after 3 days, and therefore not subjected to further cell-induced reorientation after this time-frame. The small differences are caused by the additional compaction of the collagen gels perpendicular to the tissue constraints occurring during the last 3 days.

Interestingly, the hypotheses of contact guidance and steric hindrance resulted in qualitatively different SF organizations for uniaxially constrained gels with immediate cyclic straining. In particular, the model with steric hindrance predicted SF organizations similar to the ones obtained after 3 days of remodeling, both for low and high collagen densities. This occurs because cells are subjected to the same mechanical environment as experienced during the first 3 days, and therefore they conserve their organization, irrespective of the collagen content. On the other hand, the computational model with contact guidance predicted that, during the last 3 days of remodeling, collagen provides cells with topographical cues that enhance the cross-like shaped organization obtained during the first 3 days, especially for high cyclic strain amplitudes and high collagen density. The differences between the two models are particularly visible when comparing Fig. 8AE with Fig. 8AK: in these cases, steric hindrance predicts that SFs mainly align along the constraint, while the SF alignment resulting from contact guidance is strongly aligned in between the constraint and the free edge of the tissue. Nevertheless, note that both contact guidance and steric hindrance are able to predict qualitatively the experiments of Obbink-Huizer *et al*.^29^, who cyclically stretched uniaxially constrained collagen gels with low collagen density and observed SF alignment in the constrained and stretched direction.

Figure 8BA–BR provide an overview of the results obtained for uniaxially constrained collagen gels with a delayed cyclic stretch. Overall, all computational simulations predicted SF distributions mainly aligned in the direction of the tissue constraint and applied cyclic stretch, with different degrees of alignment. Larger degrees of alignment were observed for tissues with high collagen density (Fig. 8BD–BF and BJ–BL), due to the remodeling inhibition (steric hindrance) or topographical cues (contact guidance) provided by the collagen aligned along the same direction (Fig. 8BP–BR). On the other hand, for low collagen density, SFs exhibited an organization comparable to the ones observed in case of uniaxial cyclic stretch after 3 days (Fig. 8BA–BC and BG–BI). In this case, it appeared that contact guidance slightly increased the alignment of cells towards the constraint (Fig. 8BA–BC).

## Discussion

Recent studies have demonstrated that extracellular collagen can affect cellular reorientation in response to cyclic strain^12,14,15^. Existing computational models cannot predict this phenomenon because they do not consider the combined effects of collagen and cyclic strain on cellular reorientation. To overcome this limitation, we extended a recently developed computational model (including only mechanical stimuli) by considering two hypotheses on how collagen influences cellular orientation: collagen contributes to cell alignment by providing topographical cues (contact guidance); collagen causes a spatial obstruction for cellular reorientation (steric hindrance). Furthermore, an evolution law for the cell-induced reorientation of collagen was proposed. Bi- and uniaxially constrained cell-populated collagen gels undergoing cyclic strain were simulated to test our computational framework and get more insight into the interplay between cells and collagen. Considering either contact guidance or steric hindrance, the simulations could predict the cellular and collagen orientation observed in previous experiments. Furthermore, the simulations identified untested experimental conditions such that the hypotheses of contact guidance or steric hindrance lead to qualitatively different computational results. In particular, for uniaxially constrained high-density collagen gels undergoing 10% cyclic stretch, contact guidance determined a cross-like cellular distribution, while steric hindrance led to alignment mainly in the constrained direction (Fig. 8AE and AK).

Theoretically, performing this experiment and comparing the results with the simulations could provide valuable information for determining the dominant mechanism linking collagen to cellular (re)orientation in cell-populated collagen gels. In practice, however, such one-to-one comparison would be complicated by the following observations. The computational results for uniaxially constrained gels are strongly affected by the degree of compaction perpendicular to the free edge of the gel, which could be underestimated in our study compared to the experimental results. Larger compactions lead to higher alignment along the constraints for both SFs and collagen, and this could shift the cruciform SF distribution predicted with the hypothesis of contact guidance towards a distribution similar to the one obtained with the hypothesis of contact guidance. Furthermore, the model’s outcomes are influenced by the performance of the algorithm for the prediction of the reorientation of cells in response to mechanical stimuli. Although such algorithm is able to predict the main SF orientation in uniaxially constrained collagen gels^29^, the predicted orientation is much less anisotropic than the experimentally observed one^29^. This discrepancy may become more pronounced when coupled with the additional effects of contact guidance and steric hindrance, thereby affecting the quality of the computational predictions. Due to these reasons, we foresee that obtaining a clear-cut answer regarding the dominant mechanism responsible for the effects of collagen effect on cellular orientation, by performing the experiments under the conditions identified by the simulations, is not straightforward.

Moreover, since both mechanisms proposed in this study are motivated by experimental evidence, it is very well possible that both mechanisms play a role (simultaneously); contact guidance and steric hindrance could have synergistic effects on cellular (re)orientation. In our study, they were treated separately because, if considered together with the current hypotheses on the time-scales of their effects, steric hindrance would always dominate contact guidance. In fact, steric hindrance effects would inhibit the reorientation of cells, both in response to mechanical stimuli and contact guidance. Future studies could refine our hypotheses on the time-scales of the effects of steric hindrance and contact guidance, and on the functions chosen to model these phenomena (equations (5) and (7)). In case that the effects of contact guidance occur earlier than steric hindrance, the two hypotheses could be implemented together by multiplying equation (4) with the function defined by equation (7).

For our simulations, we also assumed affine deformations in the collagen gels. The deformations in these tissues are actually non-affine, and it has been demonstrated that, in such tissues, the local stretch is inferior to the global externally applied stretch^49^. This consideration would suggest that cells in stable high-density collagen gels do not respond to external mechanical stimuli because they are subjected to lower degrees of local stretch compared to the externally applied. However, this hypothesis does not explain the realignment of Rho-activated cells observed in the same set of experiments that was analysed in this study^15^, which confirms that cells sense the external cyclic strain, despite the high-density collagen network.

Degradation and synthesis of collagen were not included in the computational framework of this study. In the early stage after seeding collagen gels (few days), which was simulated in this study, only a small percentage of collagen is degraded^50,51^, and cells only synthetize procollagen, which is not included in the existing matrix^52^. For long-term simulations these processes could be included, for example as proposed by Loerakker *et al*.^53^. Collagen crosslinks were also not considered in our simulations, since these covalent interactions form only in the long term^9,50^. For the stabilization of the collagen network in the short term, non-covalent interactions^46^ and cellular entanglement^47^ are known to occur. The kinetics of this stabilization process is unfortunately not known. Here we simply assumed that complete stabilization is achieved 3 days after seeding, after which collagen fibers cannot be reoriented anymore and start providing cells with contact guidance or steric hindrance. Modelling this collagen stabilization as a gradual process, for example by assuming a linear increase rather than a step function, did not change the computational results.

In one of our previous studies^40^, we proposed a computational approach based on thermodynamics to simulate the remodeling and interaction of stress fibers and focal adhesion signalling for cells on cyclically strained grooved surfaces. On such surfaces, cells respond to both mechanical and topographical stimuli. Therefore, the underlying physics and biology is most likely similar for cells in cyclically strained collagen gels, simulated in the present study. Thus, this computational algorithm could in principle serve to model and simulate also the behaviour of cells in dynamically loaded collagenous tissues. Compared to the phenomenological approach proposed in the present study, the previous approach^40^ has the advantage of being based on thermodynamics. However, adopting the same theory and approach to simulate cells in cyclically stretched collagen gels is in practice impossible because of excessive computational costs. Predicting the focal adhesion dynamics and signalling requires solving highly coupled partial differential equations. For cells on cyclically stretched microgrooves (simulated in Ristori *et al*.^40^), solving these equations was possible due to the particular characteristics of the environment, which enabled some approximations and a drastic decrease of the computational cost. In contrast, for cells in collagen gels, a large number of these characteristics are not valid anymore, leading to prohibitively higher computational costs.

The present approach is relevant for the prediction and explanation of cellular (re)orientation in other collagenous tissues, such as native or tissue-engineered arteries and heart valves. *In vivo*, cells in such tissues are subjected to mechanical stimuli caused by blood circulation and are surrounded by collagen with densities even higher than *in vitro*^54^. On the one hand, steric hindrance is expected to play a major role in tissues with low porosity and pore size^44,55^. On the other hand, contact guidance is probably important in tissues with highly crosslinked and aligned collagen, where contact guidance could induce a positive feedback. In particular, directions with high density of collagen would trigger the co-alignment of cells and thus more collagen synthesis in those directions, which would in turn enhance the effects of contact guidance. This positive feedback may explain the highly anisotropic collagen architectures observed for example in adult aortic heart valves^56^.

In conclusion, in the present study we extended a previous computational model that predicted cellular reorientation in response to mechanical stimuli, by including the effects of collagen (with contact guidance or steric hindrance) and by proposing an evolution law for the cell-driven reorientation of collagen. The developed computational framework, both with contact guidance or steric hindrance, is able to predict the cellular and collagen alignment previously observed in collagen gels subjected to uniaxial cyclic strain. We expect this approach to be valuable for the prediction and explanation of cellular (re)orientation in other collagenous tissues, such as native or tissue-engineered arteries and heart valves.

## Methods

### Computational simulation of collagen gel remodeling

Collagen gels were simulated with the same time period as in the experiments^12,15,29^ (6 days). Each cell-populated collagen gel as a whole was modelled as a three-dimensional material, where the nonfibrous part was modelled as a Neo-Hookean material, while for the fibers we only considered in-plane development and reorganization, as fibers were predominantly observed in-plane in previous experimental studies^12,15,29^. Therefore, in our computational study, we hypothesized that collagen and stress fibers are also distributed in one plane throughout the entire duration of the simulation. In particular, similar to Obbink-Huizer *et al*.^57^, the constitutive equation of the cell-populated collagen gels was modelled as a mixture of cells, collagen and other isotropic constituents:

9 $${\boldsymbol{\sigma }}={{\boldsymbol{\sigma }}}_{sf}+{{\boldsymbol{\sigma }}}_{cf}+{{\boldsymbol{\sigma }}}_{mc}$$

where ${\boldsymbol{\sigma }}$ is the total Cauchy stress, ${{\boldsymbol{\sigma }}}_{sf}$ the SF stress, ${{\boldsymbol{\sigma }}}_{cf}$ the collagen fiber stress, and ${{\boldsymbol{\sigma }}}_{mc}$ takes into account the remaining isotropic components. The SF stress ${{\boldsymbol{\sigma }}}_{sf}$ was modelled by extending the computational model proposed by Obbink-Huizer *et al*.^29^ with the inclusion of the effects of collagen on SF remodeling, as explained earlier (also see more details in the supplementary information). The collagen fiber stress ${{\boldsymbol{\sigma }}}_{cf}$ was computed as in Obbink-Huizer *et al*.^57^, but considering collagen prestretch as described by Loerakker *et al*.^53^ and including a reorientation law for collagen to consider the effects of cellular forces on the initial reorganization of the developing collagen network. Finally, a compressible Neo-Hookean material model was used for the isotropic term ${{\boldsymbol{\sigma }}}_{mc}$, as in Obbink-Huizer *et al*.^29,57^ and Loerakker *et al*.^53^. For a complete overview of the equations, we refer the reader to the supplementary information. Concerning the equations used for collagen reorientation and SF remodeling, having assumed the collagen network to be stable only after 3 days after seeding, equation (3) was chosen for the first 3 days to capture the remodeling of SFs without the effects of collagen, while equations (1) and (2) were chosen to model the early collagen reorganization occurring. For the remaining time (last 3 days), collagen reorientation was inactivated due to collagen network stabilization, while equation (4) or equation (6) were used to capture the effects of contact guidance or steric hindrance, respectively. Similar to Loerakker *et al*.^53,58^, we assumed that collagen and SFs would only develop within the plane of the tissue, and the fiber directions for collagen and SFs were initially isotropically distributed with an angular resolution of 6°. Their initial directions were defined as ${\overrightarrow{e}}_{f0}^{i}=\,\cos ({\gamma }^{i}){\overrightarrow{v}}_{1}+\,\sin ({\gamma }^{i}){\overrightarrow{v}}_{2}$, where ${\overrightarrow{v}}_{1}$ and ${\overrightarrow{v}}_{2}$ are orthogonal vectors and ${\gamma }^{i}$ is the angle of each direction with respect to ${\overrightarrow{v}}_{1}$, which was chosen as the direction of the applied uniaxial cyclic strain. The commercial finite element package ABAQUS (Sassault Systèmes Simulia Corp., Providence, RI, USA) was used for the computational simulations of the collagen gels. The material behaviour was implemented in the user subroutine UMAT. To enable the simulation of relatively long time periods of tissue remodeling in a reasonable amount of computational time, we employed a numerical approach that was developed in a previous study^59^ (see supplementary information for a summary of this approach).

### Geometry and boundary conditions

Only the central inner layer of the tissues prepared in Foolen *et al*.^12,15^ was analysed in the computational simulations. For the biaxially constrained tissues, we assumed the deformation in the central part of the tissue to be affine. Given this assumption, and the fact that the remodeling of SFs and collagen is completely determined by deformations, one single hexahedral element for each gel was sufficient for its analysis (Supplementary Fig. S1). Stretching was applied biaxially to match the actual stretching values that were measured in the experiments to simulate^15^: 5.3% of stretch parallel to the cyclic stretching direction and −0.7% of stretch perpendicular for 5% stretching amplitude; 11.4% parallel and −1.6% perpendicular for 10%; and 16.9% parallel and −3.2% perpendicular for 15%. In case of static conditions, the outer edges were completely constrained.

Uniaxially constrained collagen gels undergoing cyclic stretch were also simulated. For these simulations, the same mesh as in Obbink-Huizer *et al*.^29^ was chosen (Fig. 6 and Supplementary Fig. S1). In particular, due to symmetry, only one fourth of the entire gel was considered and modelled as a square shaped material. Tissue thickness was equal to 10% of each edge, and chosen to be in the same order of magnitude to tissue thickness assessed experimentally. While two adjacent edges were constrained along their perpendicular direction due to symmetries, a third edge was constrained along its parallel direction to mimic the effects of the uniaxial constraint. Two different types of boundary conditions were applied to this same edge along its perpendicular direction, corresponding to static or dynamic conditions. For static conditions, the edge was constrained along the perpendicular direction. For dynamic conditions, the edge was cyclically displaced until reaching 5%, 10%, or 15% of the tissue’s original length, with a frequency of 0.5 Hz. Finally, the fourth edge was allowed to deform freely according to the material response over time (Supplementary Fig. S1).

### Initial conditions and model parameters

The parameters characterizing the remodeling of SFs in response to cyclic strain were taken from Obbink-Huizer *et al*.^29^ and, as considered in the same study, the SFs were assumed totally disassociated at the start of the simulations, that is to say ${\phi }_{sf}^{i}=0$ and ${\varphi }_{a}={\varphi }_{m}$ for *t* = 0, where ${\varphi }_{a}$ is the total actin volume fraction. Given that collagen self-polymerization occurs much faster than cellular orientation in collagen gels^15,60^, we neglected the self-polymerization process and we assumed that collagen is totally polymerized once the mechanical stimuli are applied to the collagen gels and it initially exhibits an isotropic distribution, such that ${\phi }_{cf}^{i}={\varphi }_{cf}/N$ for all $i=1,\mathrm{...},N$. This value was chosen to be 0.5 or 0.15 for high or low collagen densities, respectively, conserving the ratio used in the experiments. The parameters describing the effects of contact guidance and steric hindrance on SF remodeling were fitted with the experimental results. The remaining parameters present in the computational model were chosen in accordance with previous studies^58,61^ (see supplementary information for more details, and Supplementary Table S1 for a list of the parameters used).

### Post-processing of the results

The fiber directions ${\overrightarrow{e}}_{f}^{i}$ change over time due to deformations. To take this evolution into account for the representation of the results, during postprocessing ${\phi }_{cf}^{i}$ and ${\phi }_{sf}^{i}$ were multiplied by the ratio of the undeformed angle between its two neighbouring directions to the corresponding deformed angle, as in Obbink-Huizer *et al*.^29^.

### Data availability statement

The datasets generated during and/or analysed during the current study are available from the corresponding author on reasonable request.

## Electronic supplementary material

Supplementary information
