# Supplementary material for: Modelling The Combined Effects Of Collagen and Cyclic Strain On Cellular Orientation In Collagenous Tissues
Source: Sci Rep. 2018 Jun 4;8:8518. doi: 10.1038/s41598-018-26989-y (PMC5986791; doi:10.1038/s41598-018-26989-y)
Supplement: Supplementary file 1 — Supplementary information [file 41598_2018_26989_MOESM1_ESM.pdf]

SUPPLEMENTARY INFORMATION for the scientific article

MODELLING THE COMBINED EFFECTS OF COLLAGEN AND CYCLIC STRAIN ON CELLULAR ORIENTATION IN COLLAGENOUS TISSUES

T. Ristori[a,b], T.M.W. Notermans[a], J. Foolen[a,b], N.A. Kurniawan[a,b], C.V.C. Bouten[a,b], F.P.T. Baaijens[a,b], S. Loerakker[a,b,*]

[a]Department of Biomedical Engineering, Eindhoven University of Technology, PO Box 513, 5600 MB Eindhoven, The Netherlands

[b]Institute for Complex Molecular Systems, Eindhoven University of Technology, PO Box 513, 5600 MB Eindhoven, The Netherlands

[*]S.Loerakker@tue.nl

Summary of the computational model. In this study, the effects of collagen architecture on cellular remodeling were investigated by modifying and extending the computational model previously proposed by Obbink-Huizer et al.[28,56]. In particular, similar to Obbink et al.[56], the constitutive equation of the cell-populated collagen gels was modelled as a mixture of cells, collagen, and other isotropic constituents:

$$\boldsymbol{\sigma} = \boldsymbol{\sigma}_{sf} + \boldsymbol{\sigma}_{cf} + \boldsymbol{\sigma}_{mc}, \tag{S.1}$$

where $\boldsymbol{\sigma}$ is the total Cauchy stress, $\boldsymbol{\sigma}_{sf}$ the SF stress, $\boldsymbol{\sigma}_{cf}$ the collagen fiber stress, and $\boldsymbol{\sigma}_{mc}$ takes into account the remaining isotropic components, such as proteoglycans. The SF stress $\boldsymbol{\sigma}_{sf}$ was modeled by extending the computational model proposed by Obbink et al.[28], with the inclusion of the effects of collagen on SF remodeling. The collagen fiber stress $\boldsymbol{\sigma}_{cf}$ was computed as in Obbink et al.[56], but considering collagen prestretch as described by Loerakker et al.[52] and including a reorientation law for collagen to consider the effects of cellular forces on the initial reorganization of the developing collagen network. Finally, similar to Obbink et al.[28,56] and Loerakker et al.[52], the isotropic components were modelled as a compressible Neo-Hookean material. Specifically,

$$\boldsymbol{\sigma}_{mc} = \phi_{mc}\left(\kappa\,\frac{\log J}{J}\boldsymbol{I} + \frac{G}{J}\left(\boldsymbol{B} - J^{2/3}\boldsymbol{I}\right)\right), \tag{S.2}$$

where ϕ_{mc} is defined as the volume fraction of the isotropic matrix components, $J = \det(\boldsymbol{F})$, and $\boldsymbol{B} = \boldsymbol{F} \cdot \boldsymbol{F}^T$, with $\boldsymbol{F}$ the deformation gradient tensor. κ and G are the compression and shear moduli, respectively defined as $\kappa = \frac{E_{NH}}{3(1-2\nu)}$ and $G = \frac{E_{NH}}{2(1+\nu)}$, with E_{NH} and ν labeling the Young's modulus and the Poisson's ratio of the isotropic components.

In the following, the original computational models for SF stress, remodeling, collagen fiber stress, and prestretch are first summarized. Subsequently, an evolution law for collagen reorientation in collagen gels is proposed, together with the corresponding extensions of the evolution law for SF remodeling.

Original model for SF remodeling and collagen prestretch. In the computational model of Obbink-Huizer et al.[28], the total SF stress was computed as

$$\boldsymbol{\sigma}_{sf} = \frac{1}{N}\sum_{i=1}^{N} \varphi_{sf}^{i}\sigma_{sf}^{i}\vec{e}_{f}^{\,i}\vec{e}_{f}^{\,i}, \tag{S.3}$$

where N is the number of considered fiber directions, while φ_{sf}^{i} and σ_{sf}^{i} label the SF volume fraction and SF stress along the i-th direction, whose orientation in the deformed configuration is indicated by $\vec{e}_{f}^{\,i}$.

Due to the similarities of the mechanisms that determine SF and sarcomere contractions, the magnitude of σ_{sf}^{i} was computed with a simplified version of the Hill model (Hill et al. 1938):

$$\sigma_{sf}^{i} = \sigma_{max} f_{\varepsilon}(\varepsilon^{i}) f_{\dot{\varepsilon}}(\dot{\varepsilon}^{i}), \tag{S.4}$$

with σ_{max} representing the maximum SF stress, while $f_{\varepsilon}(\varepsilon^{i})$ and $f_{\dot{\varepsilon}}(\dot{\varepsilon}^{i})$ are functions defined to take into account the influence of the strain ε^{i} and strain rate $\dot{\varepsilon}^{i}$ experienced by the SFs along the i-th direction. The Green-Lagrange definition of the strain was used, such that $\varepsilon^{i} = \frac{1}{2}\left(\left(\lambda_{f}^{i}\right)^{2} - 1\right)$,

with $\lambda_f^i = \sqrt{\vec{e}_f^i \cdot \boldsymbol{F} \cdot \boldsymbol{F}^T \cdot \vec{e}_f^i}$ the global stretch along the i-th direction, characterized in the original configuration by the vector $\vec{e}_{f0}^i$. Similar to Vernerey and Farsad[30], the SF stress dependence on the strain was modelled as a summation of an active ($f_{\varepsilon,a}(\varepsilon^i)$) and a passive ($f_{\varepsilon,p}(\varepsilon^i)$) component, such that

$$f_\varepsilon(\varepsilon^i) = f_{\varepsilon,a}(\varepsilon^i) + f_{\varepsilon,p}(\varepsilon^i). \tag{S.5}$$

The active component, which represents the acto-myosin contraction, is defined as

$$f_{\varepsilon,a}(\varepsilon^i) = \exp(-(\varepsilon^i/\varepsilon_0)^2), \tag{S.6}$$

while the passive part considered the strain-hardening response of extended SFs and has the form

$$f_{\varepsilon,p}(\varepsilon^i) = \begin{cases} 0 & \text{if } \varepsilon^i < 0, \\ (\varepsilon^i/\varepsilon_1)^2 & \text{if } \varepsilon^i \geq 0. \end{cases} \tag{S.7}$$

In these definitions, the constants ε_0 and ε_1 calibrate the decrease of stress for values of strain different from zero and the increase of stress in extension, respectively. Similar to Deshpande et al.[29], it was assumed that SF stress decreases in response to fast compressions according to

$$f_{\dot{\varepsilon}}(\dot{\varepsilon}^i) = \frac{1}{1+\frac{2}{\sqrt{5}}}\left(1 + \frac{k_v\dot{\varepsilon}^i+2}{\sqrt{(k_v\dot{\varepsilon}^i+2)^2+1}}\right), \tag{S.8}$$

where k_v describes the rate of decrease of SF tension depending on the rate of fiber shortening.

The SF volume fraction φ_{sf}^i was assumed to vary over time depending on the exerted active tension, as described by:

$$\frac{d\varphi_{sf}^i}{dt} = \left(k_0^f + k_1^f \sigma_{max} f_{\varepsilon,a} f_{\dot{\varepsilon}}\right)\varphi_m - k_d \varphi_{sf}^i. \tag{S.9}$$

Here, k_0^f characterizes the basal SF formation, k_1^f its dependence on the strain and strain rate, while k_d describes the rate of dissociation. The variable φ_m is defined as the volume fraction of monomeric actin, related to the SF volume fractions by means of a conservation law of actin content:

$$\phi_a = \varphi_m + \frac{1}{N}\sum_{i=1}^N \varphi_{sf}^i, \tag{S.10}$$

where ϕ_a represents the constant total actin volume fraction. Note that, in the main text of this work, the first term on the right side of equation (S.9) was represented with the auxiliary function defined as

$$f_{mech}(\varepsilon, \dot{\varepsilon}) = \left(k_0^f + k_1^f \sigma_{max} f_{\varepsilon,a} f_{\dot{\varepsilon}}\right), \tag{S.11}$$

which was used just for notation purposes, to summarize the term of the equation embedding the effects of mechanical stimuli on SF remodeling.

Collagen fiber stress was computed as previously proposed by Loerakker et al.[52], who used a similar approach as Obbink-Huizer et al.[56], but with the inclusion of the effects of prestretch. In particular, the total collagen stress was described as

$$\boldsymbol{\sigma}_{cf} = \sum_{i=1}^N \varphi_{cf}^i \sigma_{cf}^i \vec{e}_f^i \vec{e}_f^i, \tag{S.12}$$

with σ_{cf}^i the magnitude of the collagen fiber stress and φ_{cf}^i the collagen fiber volume fraction along the i-th direction, which is related to the constant total collagen volume fraction ϕ_{cf} according to

$$\phi_{cf} = \sum_{i=1}^N \varphi_{cf}^i. \tag{S.13}$$

The collagen stress along the direction i was described as

$$\sigma_{cf}^{i} = \begin{cases} \frac{k_1 k_2}{k_3}\left(e^{k_3\left(\left(\lambda_e^i\right)^2-1\right)} - 1\right) & \text{if } \lambda_e^i < 1, \\ k_1\left(\lambda_e^i\right)^2\left(e^{k_3\left(\left(\lambda_e^i\right)^2-1\right)} - 1\right) & \text{if } \lambda_e^i \geq 1, \end{cases} \tag{S.14}$$

where k_1 and k_2 are material parameters. The small compressive stress, dependent on the parameter k_3, was introduced to increase numerical stability without affecting the results in case of prestretch[52]. The term λ_e^i is the elastic stretch, introduced to take the collagen prestretch into account and defined by partitioning the total fiber stretch λ_f^i into an elastic part λ_e^i and a growth part λ_g^i, such that

$$\lambda_f^i = \lambda_e^i \lambda_g^i. \tag{S.15}$$

The elastic stretch λ_e^i can thus be computed from equation (S.15), after having identified λ_g^i as follows. Cells were assumed to contract collagen fibers until reaching an equilibrium between stress fiber and collagen fiber stress. Therefore, the preferred collagen fiber stress is

$$\sigma_{cf,p}^i = \sigma_{sf}^i. \tag{S.16}$$

This preferred collagen fiber stress corresponds to a preferred collagen shrinkage via $\lambda_{g,p}^i$, which can be found by solving the system of equations (S.14)-(S.16) once knowing the value of λ_f^i and σ_{sf}^i. In this context, only collagen compaction was considered by constraining $\lambda_{g,p}^i \leq 1$. Given $\lambda_{g,p}^i$, it was assumed that the evolution of λ_g^i towards this (continuously updated) value can be described with

$$\frac{d\lambda_g^i}{dt} = \frac{1}{\tau_\lambda}\left(\lambda_{g,p}^i - \lambda_g^i\right), \tag{S.17}$$

with τ_λ the time constant associated with the development of collagen prestretch.

In the next paragraphs, for completeness and even though they were already introduced in the main text, we report the equations and main hypotheses proposed in this study to capture the cell-induced reorganization of unstable collagen networks and the effects that stable collagen networks have on cellular reorientation in response to cyclic strain.

Proposed model for initial collagen reorientation. We hypothesized that, when cells probe their surroundings by exerting traction forces, unstable collagen fibers are reoriented from their original orientation towards the directions with higher cellular tension. This process was modeled by assuming that the collagen fiber distribution tends to a preferred one that is equal to the distribution of SF stresses. That is to say

$$\frac{\varphi_{cf,p}^i}{\sum_{i=1}^{N} \varphi_{cf,p}^i} = \frac{\sigma_{sf}^i}{\sum_{i=1}^{N} \sigma_{sf}^i}, \tag{S.18}$$

where $\varphi_{cf,p}^i$ is the preferred collagen fiber volume fraction along a direction i. Furthermore, we assumed that the current collagen fiber volume fraction φ_{cf}^i tends to the preferred $\varphi_{cf,p}^i$ according to the first-order evolution law

$$\frac{d\varphi_{cf}^i}{dt} = \frac{1}{\tau_{cr}}\left(\varphi_{cf,p}^i - \varphi_{cf}^i\right), \tag{S.19}$$

where the parameter τ_{cr} characterizes the rate of collagen reorientation.

Effects of collagen architecture on SF remodeling. Effects of loose collagen on cellular reorientation are not likely to be significant. For this reason, we assumed that SF remodeling can be influenced by the collagen network only once this is stabilized by non-covalent interactions

and cellular crosslinks. Once stable, collagen can influence SF remodeling via (at least) two possible mechanisms, which are treated below.

Model for contact guidance. We hypothesized that collagen provides cells with topographical cues, such that SFs tend to align along directions with higher collagen densities. To model this phenomenon, we adapted equation (S.9) by assuming that higher collagen volume fractions along specific directions induce more SF formation. In particular, the SF remodeling is described by

$$\frac{d\varphi_{sf}^{i}}{dt} = \left(f_{mech}(\varepsilon^{i}, \dot{\varepsilon}^{i}) + f_{cg}(\varphi_{cf}^{i})\right)\varphi_{m} - k_{d}\varphi_{sf}^{i}, \tag{S.20}$$

where

$$f_{cg}(\varphi_{cf}^{i}) = g_{cg}\left(1 - \frac{h_1}{\exp(h_2 + h_1 - 1)}\right) \tag{S.21}$$

is a monotonically increasing function dependent on φ_{cf}^{i} that describes the increase of SF formation for increasing collagen content along the i-th direction. In the definition, g_{cg} represents the maximum effect of contact guidance on SF remodeling, while h_1 and h_2 are parameters characterizing the inflection point and the associated slope of the function f_{cg}. While the inflection point is inversely proportional with h_2, it increases with increasing values of h_1. On the other hand, the slope is directly proportional with h_2 and it decreases for increasing values of h_1.

Model for steric hindrance. We propose that, due to spatial restrictions caused by stable and high-density collagen networks, the SF remodeling potential decreases with increasing collagen density and is completely inhibited when a certain threshold density is exceeded. This mechanism was modeled by multiplying the term in the right-hand side of equation (S.9) with a monotonically decreasing function dependent on the total collagen fiber volume fraction ϕ_{cf}. Specifically, considering the effects of steric hindrance without contact guidance, SF remodeling is described by

$$\frac{d\varphi_{sf}^{i}}{dt} = \left(f_{mech}(\varepsilon^{i}, \dot{\varepsilon}^{i})\varphi_{m} - k_{d}\varphi_{sf}^{i}\right)f_{sh}(\phi_{cf}), \tag{S.22}$$

with

$$f_{sh}(\phi_{cf}) = \begin{cases} 1 - g_{sh}\phi_{cf} & \text{if } \phi_{cf} < 1/g_{sh}, \\ 0 & \text{if } \phi_{cf} \geq 1/g_{sh}. \end{cases} \tag{S.23}$$

Here, the parameter g_{sh} describes the decrease of the rate of SF remodeling for increasing total collagen volume fraction.

Analytical approximation of evolution laws. To enable the simulation of relatively long time periods of tissue remodeling in a reasonable amount of computational time, the ordinary differential equations describing the evolution of SFs, collagen, and prestretch (equations (S.9), (S.17), (S.19), (S.20)) were solved by using a previously developed analytical approximation[58]. Briefly, under certain conditions, given a system of ordinary differential equations of the form

$$\dot{x}(t) = f(t)x(t) + g(t), \tag{S.24}$$

where $x: \mathbb{R} \to \mathbb{R}^n$ is the solution of the system of equations of dimension $n \in \mathbb{N}$, while $f: \mathbb{R} \to \mathbb{R}^{n \times n}$ and $g: \mathbb{R} \to \mathbb{R}^{n \times n}$ are periodic functions, it can be shown that the solution $x(t)$ tends to a function $x_\infty(t) = \lim_{t\to\infty} x(t)$ that can be approximated with

$$x_\infty(t) \approx -\bar{f}^{-1}\bar{g}, \tag{S.25}$$

with the overbar denoting the average of the functions over their period. For example, by applying this method to equation (S.20) for contact guidance, we can determine that SFs tend to a preferred SF distribution $\varphi_{sf,p}^{i}$ such that

$$\varphi_{sf,p}^{i} = \frac{\bar{a}_i}{\sum_{j=1}^{N} \frac{\bar{a}_j}{N} + k_d} \phi_a, \tag{S.26}$$

with

$$\bar{a}_i = \frac{1}{T} \int_0^T \left[k_0^f + k_1^f \sigma_{max} f_{\varepsilon,a}(\varepsilon^i) f_{\dot{\varepsilon}}(\dot{\varepsilon}^i) + f_{cg}(\varphi_{cf}^i)\right] dt, \tag{S.27}$$

With the analytical approximation for the asymptotic solution of equation (S.20), we assumed that SFs tend to this preferential distribution according to

$$\frac{d\varphi_{sf}^i}{dt} = \frac{1}{\tau_{sf}} \left(\varphi_{sf,p}^i - \varphi_{sf}^i\right), \tag{S.28}$$

with τ_{sf} characterizing the remodeling rate.

While this approach was successfully applied for equations (S.9), (S.17), (S.19), and (S.20), if this is applied to solve equation (S.22), no effects of steric hindrance would be observed. This occurs because, according to this hypothesis, collagen only affects the rate of SF remodeling; it does not influence the asymptotic SF distribution, so $\varphi_{sf,p}^i$ is unaffected. Therefore, to solve equation (S.22) while considering the effects of steric hindrance, we chose a different approach. While the asymptotic SF distribution was still identified with the analytical approximation (equation (S.25)), to take the effects of collagen on the remodeling rate into account, we assumed that SFs tend to the preferential distribution according to:

$$\frac{d\varphi_{sf}^i}{dt} = \frac{f_{sh}(\phi_{cf})}{\tau_{sf}} \left(\varphi_{sf,p}^i - \varphi_{sf}^i\right), \tag{S.29}$$

where $f_{sh}(\phi_{cf})$ is defined in equation (S.23).

Due to these analytical approximations, it was possible to consider the dynamic nature of the cyclic strain applied to the tissues and its influence on collagen and SF remodeling without effectively applying the complete stretching profile on the tissues for 6 consecutive days. Particularly, only a subset of the total amount of load cycles was applied, while the major part of the dynamic load cycles was incorporated via the analytical approximation[58] (Fig. S2).

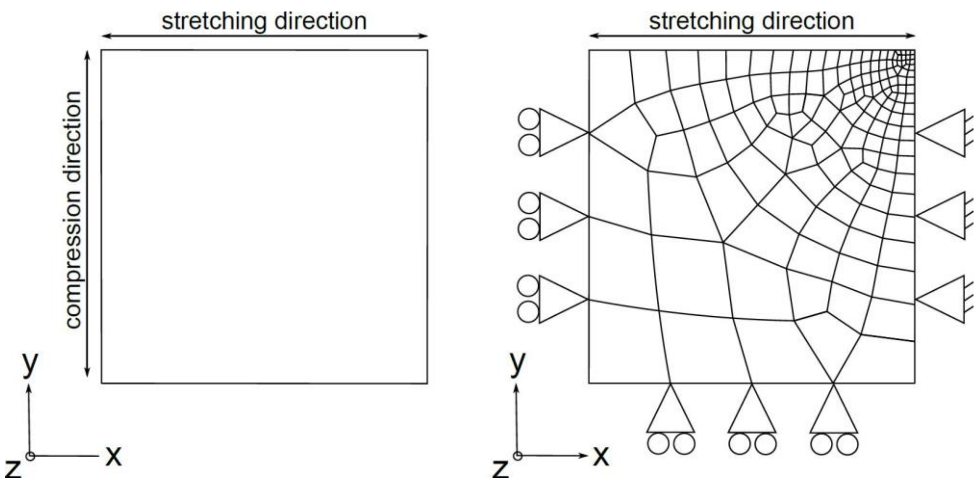

Figure S1: Mesh and boundary conditions for the simulations of collagen gels which were biaxially (left) or uniaxially (right) constrained, and uniaxially cyclically stretched.

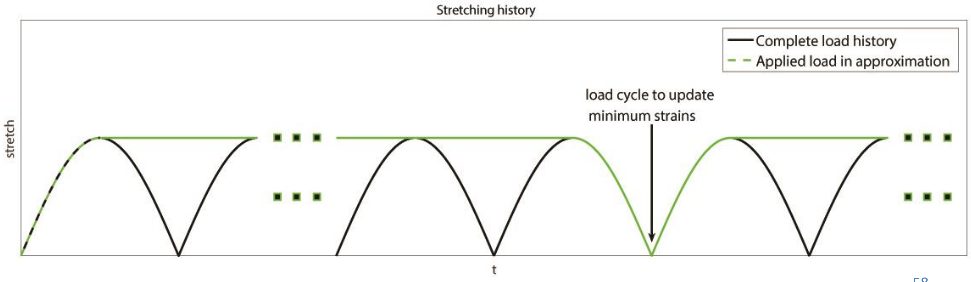

Figure S2: Profile of the loading cycle used in the approximation, adapted from Ristori et al.[58].

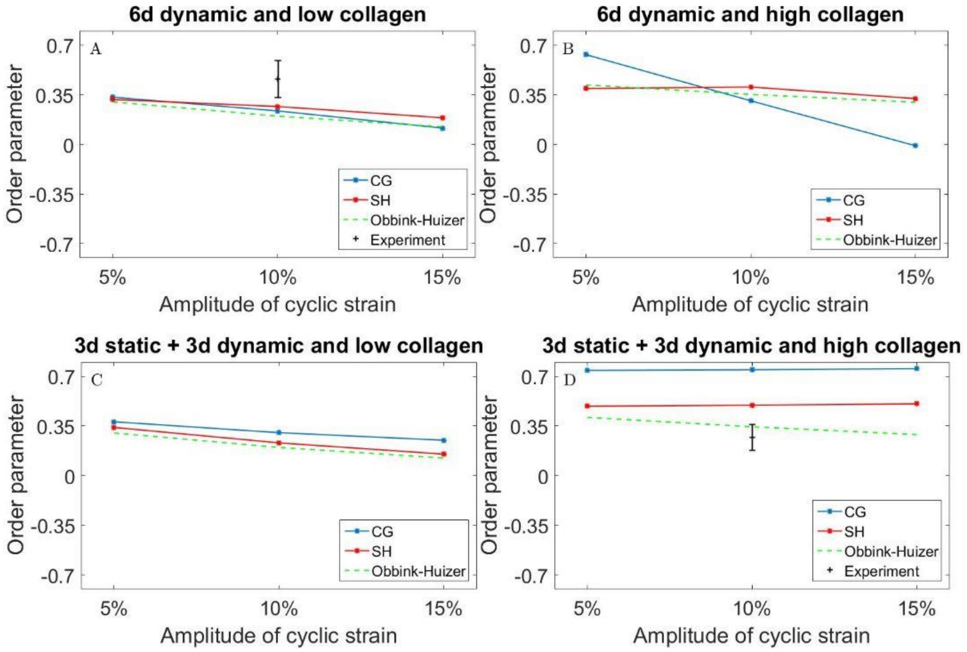

Figure S3: Comparison of the order parameters of the SF distributions in uniaxially constrained collagen gels, as predicted by the two extended computational models (blue for contact guidance, red for steric hindrance) and the original model of Obbink-Huizer et al.[28] (green), with low (A, C) and high (B, D) collagen densities. When available in the literature[12,28], previous experimental results were reported by showing the mean order parameter and the standard deviation (black).

Table S1: Parameter set for computational simulation of collagen gels

Model component	Parameter	Value	Reference
SFs	ϕ_a	0.05	[28]
	σ_{max}	200 kPa	[28]
	ε_0	0.12 (−)	[28]
	ε_1	0.17 (−)	[28]
	k_v	50 s	[28]
	k_0^f	$1.5 \cdot 10^{-6} \mathrm{s}^{-1}$	[28]
	k_1^f	$7.0 \cdot 10^{-7} \mathrm{s}^{-1} \mathrm{Pa}^{-1}$	[28]
	k_d	$1.0 \cdot 10^{-3} \mathrm{s}^{-1}$	[28]
Contact guidance	g_{cg}	2 (−)	
	h_1	500 (−)	
	h_2	300 (−)	
Steric hindrance	g_{sh}	2 (−)	
Collagen fibers	ϕ_{cf}	0.5 or 0.15 (−)	
	k_1	22 kPa	[60]
	k_2	7.5 (−)	[60]
	k_3	100 (−)	[52]
Remodeling rates	τ_{sf}	5 min	[57]
	τ_λ	1 h	[57]
	τ_{cr}	1 h	
Isotropic components	ϕ_{mc}	0.45 or 0.8 (−)	
	E	30 kPa	
	ν	0.3	[57]
